# The perceived neighborhood environment is associated with health-enhancing physical activity among adults: a cross-sectional survey of 13 townships in Taiwan

**DOI:** 10.1186/s12889-019-6848-4

**Published:** 2019-05-07

**Authors:** Chi-Chen Chiang, Shu-Ti Chiou, Yuan-Mei Liao, Yiing Mei Liou

**Affiliations:** 1 Department of Nursing, School of Nursing, National Yang-Ming University, Taipei, Taiwan; 2 Department of Nursing, Chang Jung Christian University, Tainan, Taiwan; 3 Center for Quality Management, Cheng Hsin General Hospital, Taipei, Taiwan; 4 Institute of Public Health, School of Medicine, National Yang-Ming University, Taipei, Taiwan; 5 Clinical Institute of Nursing, School of Nursing, National Yang-Ming University, Taipei, Taiwan; 6 Institute of Community Health Care, School of Nursing, National Yang-Ming University, 155, Li-Nong St., Sec. 2, Pai-Tou, Taipei, 112 Taiwan, Republic of China; 7 School Health Research Center, National Yang-Ming University, Taipei, Taiwan

**Keywords:** Neighborhood environment, Built environment, Environmental factor, Physical activity, Health-enhancing physical activity, International physical activity questionnaire, Multinomial logistic regression

## Abstract

**Background:**

Many environmental factors have been associated with physical activity. The environment is considered a key factor in terms of the rate of engagement in physical activity. This study examined the perceived effect of environmental factors on different levels of health-enhancing physical activity among Taiwanese adults.

**Methods:**

Data were collected from 549 adults aged at least 18 years from the northern, central, southern and eastern regions of Taiwan. Physical activity was measured using the International Physical Activity Questionnaire (IPAQ) showcard version, and participants were divided into three categories: those who performed low-, moderate-, or high-levels of physical activity, as suggested by the IPAQ scoring protocol. The perceived neighborhood environment in relation to physical activity was adapted from the Physical Activity Neighborhood Environment Scale. A multinomial logistic regression was conducted to ascertain associations between individual perceptions of the neighborhood environment and different physical activity levels.

**Results:**

Respondents who perceived their neighborhood environment as having easy access to services and stores, and higher traffic safety were more likely to be moderate level of physical activity (odds ratio [OR]: 1.90, 95% confidence interval [CI]: 1.07–3.37; OR: 1.77, 95% CI: 1.12–2.80). The perception of having easy access to services and stores and seeing many physically active people in the neighborhood were both positively associated with a high level of physical activity (OR: 2.25, 95% CI: 1.01–5.01; OR: 2.40, 95% CI: 1.11–5.23).

**Conclusions:**

Different perceived neighborhood environmental factors were associated with moderate and high levels of physical activity, respectively. These findings highlight the importance of an activity-friendly neighborhood environment to stimulate engagement in physical activity among adults in Taiwan. Therefore, policies and programs should focus on improving friendliness and diversity in neighborhoods to facilitate individuals’ transitions from inactive to active lifestyles.

**Electronic supplementary material:**

The online version of this article (10.1186/s12889-019-6848-4) contains supplementary material, which is available to authorized users.

## Background

Many studies have confirmed the benefits of regular physical activity on human health [1–4]. Middle-aged adults who regularly engage in physical activity for more than 3 h per week have been reported to be less likely to develop metabolic syndrome compared with physically inactive people [5, 6]. Physical inactivity is a key determinant of health and the development of chronic diseases. The World Health Organization (WHO) stated that inactivity is one of the top 10 leading global causes of mortality and disability worldwide, accounting for approximately 2 million deaths per year [7]. Similarly, according to the top 10 causes of death reported by the Ministry of Health and Welfare of Taiwan in 2016, six of these causes, including cancer, heart disease, cerebral vascular disease, and high blood pressure, are related to physical inactivity [8]. In a study evaluating physical activity prevalence across 20 countries, the rate of adults aged 18–65 years engaging in high level of physical activity in Taiwan was 24.8%, ranking second to last overall. The rate of physical inactivity was 42.3% in Taiwan which was the third highest among the ranked countries [9].

Previous studies have investigated the relationship between physical activity and the perceptions of the neighborhood environment in high-income countries, such as North America, Western Europe, and Australia [10–14]; however, few related studies have been conducted in Asia [15, 16]. Marked differences in living conditions and cultures exist across countries, and such differences could lead to differences in the degree of association between an environment and its residents’ level of physical activity. According to research findings, there are known relationships between the environment and total, domain-specific and intensity-specific physical activity [10–14, 17–19].

Several studies have argued that the attributes of a neighborhood environment, including residential density, population density, land use, street connectivity and sidewalks, are closely associated with various types of physical activity (PA) [17, 20–22]. In addition, physical activity benefits physical health, as stated in health-enhancing physical activity guidelines and by the WHO. The perception of a neighborhood environment plays a key role in the relationship between physical activity and environmental factors; generally, people’s perceptions and explanations of their surroundings affect their engagement in physical activity [23], and perceptual measures can be used to assess potentially key factors such as aesthetics and sense of safety that cannot be measured objectively. Higher perceived environmental factor scores have been shown to be associated with higher levels of leisure- time physical activity [24]. Thus, the relationship between the environment and different physical activity levels is complex [25]. However, the relationship between the environment and physical activity has been insufficiently investigated to draw holistic conclusions in Taiwan. This study explored the association between perceived environmental factors and different levels of physical activity among adults from 13 townships in four counties in Taiwan.

## Methods

### Design and participants

This study adopted a cross-sectional research design. Data were collected from 549 adults aged at least 18 years. To distinguish between geographical locations, these data were obtained from 13 townships from four countries in northern, central, southern and eastern Taiwan in 2012. Participants were selected using the proportional quota sampling. The design was based on the population structure of each township in terms of gender and age. Reference data came from the Taiwan population census in 2010. Inclusion criteria for participants were contacted and invited to participate in the study and being at least 18 years old and completing the structured questionnaire.

### Measurements

#### Physical activity

The International Physical Activity Questionnaire (IPAQ) showcard version (see Additional file 1) was adapted from the Taiwanese version of the IPAQ’s self-administered short and long versions [26] and was used to calculate the physical activity time, sitting time and sleeping time per week. The IPAQ showcard version included 11 items, including the time spent engaging in vigorous and moderate physical activity as well as sitting and sleeping on weekdays and weekend days in the preceding 7 days. In addition, the IPAQ showcard version contains four images and simple text to present examples of various physical activities with different levels of intensity (vigorous physical activity, moderate physical activity, walking, and sitting) in work, transportation, housework, and recreation domains. In this study, the participants were asked to recall the number of days, hours, and minutes they engaged in physical activity at each level of intensity combined with the text questionnaire and figures. The content validity index of the IPAQ showcard version was 0.95. The pretest and posttest concurrent validity values were 0.916–0.960 and 0.916–0.998, respectively, demonstrating enhanced healthy physical activity. The test–retest reliability was 0.478–0.683, the criterion-related validity was 0.192–0.405, and a *Z* test revealed no significant differences between the IPAQ showcard version and IPAQ self-administered short version. Their reliability and validity were better than those of the IPAQ self-administered short version [27]. IPAQ data were processed according to the IPAQ protocol (accessible at http://www.ipaq.ki.se) [28]. The metabolic equivalents (METs) of vigorous-intensity activity, moderate-intensity activity, and walking were 8.0, 4.0, and 3.3, respectively. MET-minutes per week were calculated as the MET intensity multiplied by the number of minutes of each activity over the preceding 7 days. The participants were divided into three categories: those who performed a low level, moderate level or high level of physical activity, as proposed in the IPAQ scoring protocol [28].

#### Perceived neighborhood environments

The questionnaire consisted of 12 items; the measures for the perceived neighborhood environment in relation to physical activity (see Additional file 2) were adopted from the Physical Activity Neighborhood Environment Scale [29]. To assess their perceptions of their neighborhood environments, the participants were asked to express their perception of their neighborhood within a 10–15 min walk from their residence on a 4-point Likert scale ranging from strongly disagree to strongly agree in regard to factors such as service and store access, traffic stop access, recreational facilities access, walking infrastructure access, walking infrastructure quality, bike lanes, crime safety, street connectivity, traffic safety, air pollution, and aesthetics, as well as seeing physically active people. Their responses were then recoded; “strongly disagree” and “disagree” were recoded as “0,” indicating strong disapproval of the perceived neighborhood environment in relation to physical activity. In contrast, positive responses were recoded as ‘1.’

#### Demographic variables

Demographic data included sex, age groups (18–34, 35–44, 45–64, and ≥ 65 years old), education level (below junior high school, senior high school, and above college and university), body mass index (BMI), and urbanization degree. A total 359 townships in Taiwan were stratified into seven degrees of urbanization according to the standard published by Taiwan’s National Health Research Institute (“1” indicated most urbanized and “7” indicated least urbanized) [30]. The main principles used to define township urbanization were the on population density (people/km^2^), the percentage of people with an educational level of college or above, the percentage of elderly people (older than 65 years), the percentage of agricultural workers, and the number of physicians per 100,000 people. The seven degrees of urbanization in townships were subsequently recoded into two categories, namely, urban and rural. Residential areas located in clusters of 1–3 were categorized as urban and the others were categorized as rural.

### Statistical analysis

Chi-square statistics were calculated to examine the relationship between demographic data and perceived neighborhood environments with respect to physical activity. One-way ANOVA was conducted to differentiate between neighborhood environments according to the three levels of physical activity (low, middle, and high) based on the amounts of physical activity in the preceding 7 days. The multinomial logistic regression was performed to determine the association between neighborhood environments and different physical activity levels. The participants were grouped into three physical activity levels (low, moderate and high). We used the low level of physical activity as the reference group and an odds ratio (OR) with a 95% confidence interval (CI) was used to determine the association between the neighborhood environments and physical activity. Univariate analysis was performed to determine the important environmental factors. All variables with *p* < 0.05 in the univariate analysis and variables that were published in related studies were entered into model 1. Model 2 was adjusted by sex, age, education level, and urbanization to examine the association found in model 1. Statistical analyses of each perceived neighborhood variable were conducted using SPSS version 20.0 (SPSS Inc., Chicago, IL, USA), and *p* < 0.05 was considered statistically significant.

## Results

### Sample and demographic data

The demographic data of the participants are shown in Table 1. The average age of the participants was 48.3 years [standard deviation (*SD)* = 17.5], and most participants were women (64.2%). Regarding educational level, most of the participants had graduated from senior high school (49.4%). Of the participants, 45.3% were overweight or obese. The percent of participants that lived in urban or rural areas were 70.0 and 30.0%, respectively. Using a chi-square test, we examined differences in the basic attributes of the three groups of PA. In this study, the demographic data were not significantly different across the three physical activity groups.

**Table 1:** Descriptive characteristics by different levels of physical activity (*N* = 549)

Items	Total	Low level	Moderate level	High level	*X* ^*2a*^	*P* ^*b*^
	*n* (%)	*n* (%)	*n* (%)	*n* (%)		
Sex					0.099	.952
Male	195 (35.8)	113 (35.3)	54 (36.5)	28 (36.8)		
Female	349 (64.2)	207 (64.7)	94 (63.5)	48 (63.2)		
Age					11.619	.071
18–34	141 (26.0)	95 (29.9)	29 (19.5)	17 (22.4)		
35–49	151 (27.8)	90 (28.3)	41 (27.5)	20 (26.3)		
50–64	151 (27.8)	74 (23.2)	49 (32.9)	28 (36.8)		
≥ 65	100 (18.4)	59 (18.6)	30 (20.1)	11 (14.5)		
Education					2.225	.694
< Junior high school	117 (21.5)	75 (23.4)	26 (17.4)	16 (21.1)		
Senior high school	269 (49.4)	154 (48.1)	78 (52.3)	37 (48.7)		
> College	159 (29.2)	91 (28.4)	45 (30.2)	23 (30.3)		
BMI^**c**^					0.373	.830
< 24	299 (54.7)	175 (54.2)	80 (54.1)	44 (57.9)		
≥ 24	248 (45.3)	148 (45.8)	68 (45.9)	32 (42.1)		
Urbanization^**d**^					0.047	.977
Urban	383 (70.0)	224 (69.8)	105 (70.0)	54 (71.1)		
Rural	164 (30.0)	97 (30.2)	45 (30.0)	22 (28.9)		

^a^Results from the chi-square for the differences between low, moderate, and high levels of physical activity

^b^There were no significant differences between various physical activity levels with respect to demographic data (*p* > 0.05)

^c^Body mass index (BMI) is a measure of body fat based on height and weight

^d^Seven degrees of urbanization in townships by the standard of Taiwan’s National Health Research Institute were subsequently recoded into two categories, namely, urban and rural

### Perceived neighborhood environment

The results of the participants’ perceived environmental factors are listed in Table 2. Each item was related to the current environment within a 10–15 min walks of their residences. The participants provided their perceptions of the neighborhood environment. During data processing, “agree” and “strongly agree” were combined for the analysis of consistency between the item and the current environment. “Disagree” and “strongly disagree” were combined for the measurement of inconsistency between the item and the environment. As presented in Table 2, statistically significant differences were observed between low, moderate, and high levels of physical activity and perceived environmental variables in the neighborhood, including services and stores access, walking infrastructure access, walking infrastructure quality, street connectivity, traffic safety, seeing many physically active people, as well as aesthetics, with *p* values less than 0.05.

**Table 2:** Relationships between perceived environmental factors and different levels of physical activity

Items	Low level	Moderate level	High level	*X* ^*2 a*^	*P* ^*b*^
	*n* (%)	*n* (%)	*n* (%)		
Access to services and stores					
No	94(29.3)	30 (20.0)	13 (17.1)	7.658	.022*
Yes	227 (70.7)	120 (80.0)	63 (82.9)		
Access to traffic stops					
No	122 (38.0)	64 (42.7)	23 (30.3)	3.300	.192
Yes	199 (62.0)	86 (57.3)	53 (69.7)		
Access to recreational facilities					
No	69 (21.5)	24 (16.0)	8 (10.5)	5.745	.057
Yes	252 (78.5)	126 (84.0)	68 (89.5)		
Access to walking infrastructure					
No	177 (56.5)	67 (46.5)	29 (39.2)	9.108	.011*
Yes	136 (43.5)	77 (53.5)	45 (60.8)		
Quality of walking infrastructure					
No	107 (53.2)	51 (46.8)	17 (31.5)	8.171	.017*
Yes	94 (46.8)	58 (53.2)	37 (68.5)		
Access to bike lanes					
No	200 (62.5)	93 (62.0)	50 (66.7)	.530	.767
Yes	120 (37.5)	57 (38.0)	25 (33.3)		
Safety from crime					
No	173 (54.1)	71 (47.3)	40 (52.6)	1.866	.393
Yes	147 (45.9)	79 (52.7)	36 (47.4)		
Street connectivity					
No	102 (32.2)	40 (26.7)	14 (18.7)	5.853	.054
Yes	215 (67.8)	110 (73.3)	61 (81.3)		
Traffic safety					
No	149 (48.5)	47 (31.5)	24 (32.4)	14.846	<.001***
Yes	158 (51.5)	102 (68.5)	50 (67.6)		
Air pollution					
No	179 (56.1)	76 (50.7)	43 (56.6)	1.350	.509
Yes	140 (43.9)	74 (49.3)	33 (43.4)		
Seeing many physically active people in the neighborhood					
No	117 (36.8)	39 (26.0)	14 (18.7)	11.986	.002**
Yes	201 (63.2)	111 (74.0)	61 (81.3)		
Aesthetics					
No	127 (39.8)	42 (28.0)	20 (26.7)	8.781	.012*
Yes	192 (60.2)	108 (72.0)	55 (73.3)		

^a^Results from the chi-square for the differences between low, moderate, and high levels of physical activity

^b^Statistically significant: *P < 0.05, **P < 0.01, ***P < 0.001

### Self-report of physical activity

**Fig. 1:**
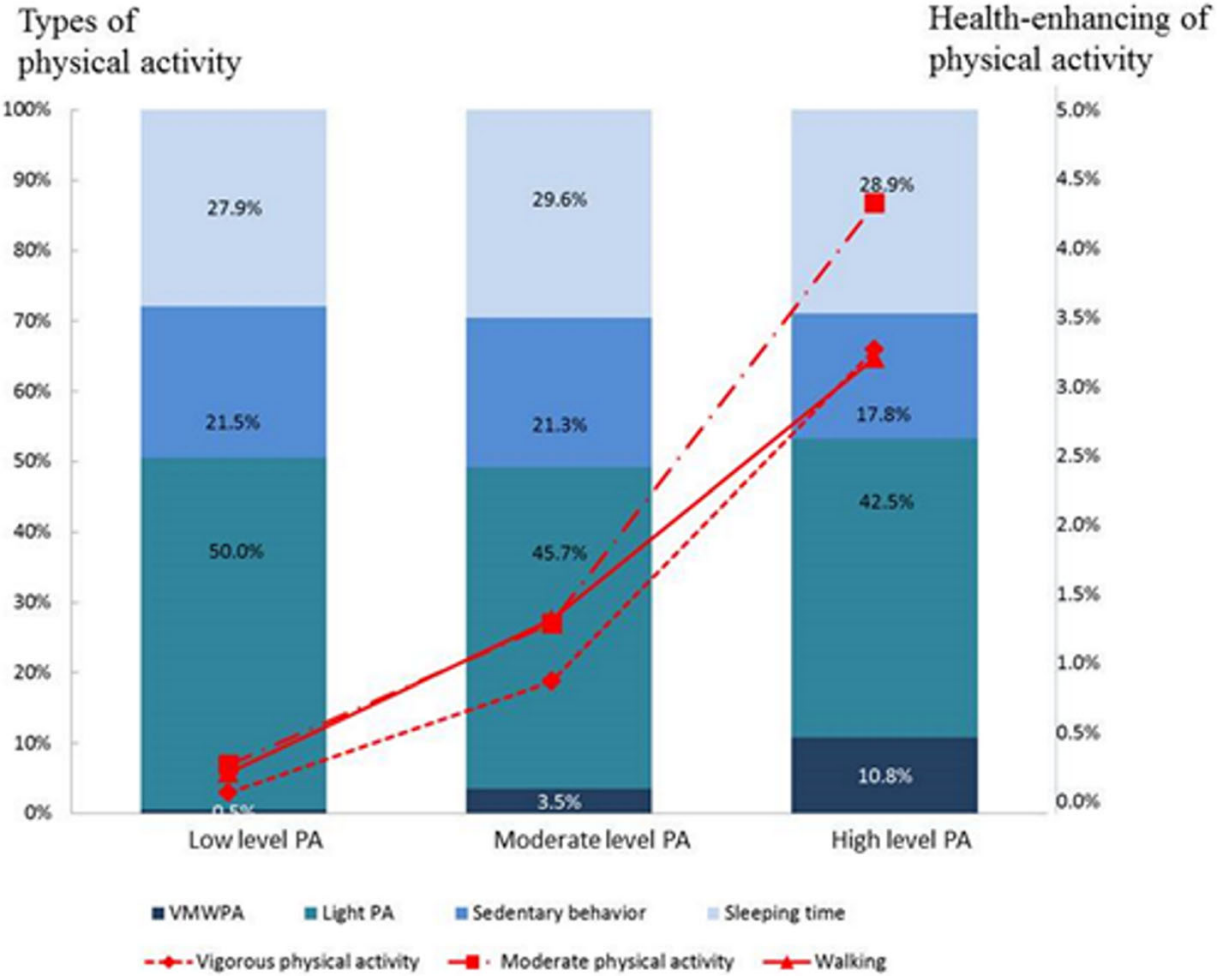
The distribution for different types and amounts of HEPA. Note: The bar chart refers to types of physical activity (represented by different colors), including durations of HEPA (sum of vigorous and moderate physical activity as well as walking), light physical activity, sitting, and sleeping. In addition, the line graph illustrates the percentages of health-enhancing vigorous and moderate physical activity and walking. Scheffe’s post hoc tests revealed that the participants in high-level physical activity group spent significantly more time in vigorous physical activity, moderate physical activity, and walking compared to moderate-level group and low-level group (*p* < 0.001)

According to the IPAQ showcard version, participants spent time engaging in vigorous-intensity activities, moderate-intensity activities, walking, sitting, and sleeping in the preceding 7 days for 72.1 ± 166.3 min, 113.0 ± 222.2 min, 83.9 ± 168.1 min, 2103.8 ± 1157.7 min, and 2870.2 ± 826.0 min, respectively. Figure 1 provides an overview of the amounts of physical activity performed by the participants. The participants were divided into three levels of physical activity. The bar chart refers to types of physical activity (represented by different colors), including the duration of health-enhancing physical activity (HEPA) (sum of vigorous and moderate physical activity as well as walking), light physical activity, sitting, and sleeping. In addition, the line graph illustrates the percentages of health-enhancing vigorous and moderate physical activity and walking. For groups performing low-, moderate-, and high-levels of physical activity, the percentages of HEPA were 0.5, 3.5, and 10.8%, respectively. One-way ANOVA was conducted on each group to determine differences between the groups in the amount of physical activity in the preceding 7 days. Significant differences were observed among the three groups with respect to the amount of vigorous and moderate physical activity, as well as walking (F = 189.7, *p* < 0.001; F = 177.0, *p* < 0.001; F = 153.8, *p* < 0.001; F = 3.1, *p* = 0.044, respectively). However, sleeping time was not significantly different between the low, moderate, and high physical activity levels (F = 2.1, *p* = 0.118). Scheffe’s post hoc tests revealed that the participants in high-level physical activity group spent significantly more time in vigorous physical activity, moderate physical activity, and walking compared to moderate-level group and low-level group (p < 0.001). Additionally, the participants in the high-level physical activity group engaged in more vigorous physical activity (3.3%), moderate physical activity (4.3%), and walking (3.2%) than those in the moderate-level group (corresponding percentages of 0.9, 1.3, and 1.3%, respectively) or low-level group (corresponding percentages of 0.1, 0.3, and 0.2%, respectively).

### Association between the neighborhood environment and physical activity

Table 3 presents the results of the series of multinomial logistic regression models examining the association between perceived neighborhood environment and physical activity. The perceived environmental factors were considered, including services and stores access, recreational facilities access, walking infrastructure, traffic safety, and aesthetics, as well as seeing many physically active people in the neighborhood. In model 1, only one perceived environmental factor was found to be significantly associated with moderate level of physical activity. The probability of a moderate level of physical activity being met was significantly related to perceived traffic safety (OR: 1.78, 95% CI: 1.14–2.76).

**Table 3:** Multinomial logistic regression analysis for the association between the neighborhood environment and physical activity among adults in Taiwan

	Model 1^c^		Model 2^d^	
	Moderate level PA	High level PA	Moderate level PA	High level PA
	OR^a^ (95% CI)^b^	OR (95% CI)	OR (95% CI)	OR (95% CI)
Perceived environmental factors				
Access to services and stores	1.63 (0.96–2.78)	2.02 (0.94–4.34)	1.90 (1.07–3.37)*	2.25 (1.01–5.01)*
Access to recreational facilities	0.86 (0.47–1.56)	1.12 (0.47–2.67)	0.72 (0.38–1.33)	0.90 (0.37–2.20)
Access to walking infrastructure	1.07 (0.69–1.66)	1.36 (0.77–2.41)	1.08 (0.68–1.70)	1.33 (0.73–2.42)
Traffic safety	1.78 (1.14–2.76)*	1.57 (0.88–2.80)	1.77 (1.12–2.80)*	1.61 (0.88–2.94)
Seeing many physically active people	1.21 (0.72–2.04)	1.68 (0.82–3.44)	1.38 (0.80–2.39)	2.40 (1.11–5.23)*
Aesthetics	1.31 (0.79–2.17)	1.04 (0.53–2.02)	1.28 (0.78–2.16)	0.98 (0.49–1.94)
Demographic data				
Sex				
Male			1.28 (0.76–2.16)	1.35 (0.76–2.39)
Female			1	1
Age (years)				
18–34			0.40 (0.19–0.85)*	0.76 (0.27–2.12)
35–49			0.63 (0.32–1.25)	0.91 (0.35–2.37)
50–64			1.12 (0.59–2.12)	1.99 (0.85–4.66)
≥ 65			1	1
Education level				
< Junior high school			0.40 (019–0.83)*	0.64 (0.26–1.55)
Senior high school			0.81 (0.49–1.35)	0.65 (0.34–1.26)
> College			1	1
Urbanization				
Urban			0.61 (0.37–1.01)	0.71 (0.36–1.37)
Rural			1	1
AIC^e^	254.9		729.2	
Nagelkerke’s R^2^	6.5%		11.7%	

The reference group is the group with a low level of physical activity

Statistically significant: *P < 0.05

^a^*OR* odds ratio

^b^*95% CI* 95% confidence interval

^c^Model 1 is the unadjusted model

^d^Model 2 is adjusted for sex, age, education level, and urbanization

^e^*AIC* Akaike’s information criterion

After controlling for sex, age, education level, and urbanization in model 2, respondents who perceived their neighborhood environments as having easy access to services and stores, not only engaged in moderate level of physical activity but were also more likely to be highly active compared with those with low physical activity level (OR: 1.90, 95% CI: 1.07–3.27; OR: 2.25, 95% CI: 1.01–5.01, respectively). In addition to easy access to services and stores, traffic safety was more likely to be engaging in a moderate level of physical activity (OR: 1.77, 95% CI: 1.12–2.80), and seeing many physically active people in the neighborhood was more likely to result in participants being highly physically active (OR:2.40, 95% CI: 1.11–5.23).

Regarding the model fitting information, in model 1, the Akaike’s information criterion (AIC) was 254.9. Pearson’s X^2^ and G^2^ values and standard deviation were 92.27 (*p* = 0.788) and 99.15 (*p* = 0.616), respectively. In addition, Nagelkerke’s R^2^ was 0.065. In model 2, the AIC was 729.2; Pearson’s X^2^ and G^2^ values and standard deviation were 674.75 (*p* = 0.298) and 590.74 (*p* = 0.968), respectively; and Nagelkerke’s R^2^ was 0.117.

## Discussions

1. Group divisions based on physical activity were appropriate

According to research on different physical activity levels, when people recall their engagement in physical activity, they tend to overestimate the time spent engaging in physical activity, particularly in groups with high activity levels [31–33]. Therefore, the IPAQ showcard version was used to measure amounts of physical activity, and the questionnaire included images and simple text to represent various types of vigorous physical activity, moderate physical activity, walking, and sitting. Furthermore, this version helped the participants in this study understand the questions and reduced the overestimation of their level of physical activity. Participants were divided into three categories representing low, moderate, and high levels of physical activity as suggested by the IPAQ scoring protocol. In terms of vigorous and moderate physical activity and walking, the amount of physical activity increased with the degree of physical activity performed by the groups. Furthermore, separating physical activity into three categories was an appropriate and sensitive method of categorizing participants that allowed researchers to analyze the relationship between various levels of physical activity and perceived neighborhood environments.

2. Safe neighborhoods with diverse shops enhance people’s levels of physical activity

According to the findings of this study, perceived environmental factors of a neighborhood, including services and stores access and traffic safety influenced the daily life activities of the group reporting a moderate level of physical activity. In other words, environmental factors related to daily life help to promote people’s activities. Similarly, according to the research findings of one study, access to services and stores, and traffic safety in a neighborhood positively influenced various types of physical activity [34–36]. According to Arngo’s (2013) systematic review, the number of accessible services and stores and the perception of safety in the neighborhood were positively correlated with recreational physical activity [37]. In addition, access to services and stores and perceived traffic safety positively influenced physical activity performed as transportation physical activity [38, 39]; hence, the social environment is crucial to physical activity. Security and traffic safety are two key determinants of physical activity. Traffic safety reinforces physical activity [40]. However, this finding was not consistent with that reported in some studies [41–43]. People aged 18–34 years focused on work and their propensity to practice physical activity was lower than people over 65 years of age. Generally, middle-aged or elderly people may begin to encounter chronic illnesses and retire. Due to health concerns and an increase in recreational time, the percentage of this group that practices physical activity increases. Generally, people with higher education and higher positions in the workplace have higher incomes, and they can therefore obtain more health information. Thus, they have more opportunities to engage in physical activity and more associated resources compared to those with a low education level.

According to the findings of this study, the government should devise strategies to enhance people’s level of physical activity; environmental factors significantly influenced the group that engaged in a moderate level of physical activity and their engagement in daily physical activity. According to the demands of communities, facilities and recreational spaces, such as green spaces in parks, shopping malls, and stores, should be established or improved to enhance the community’s practice of physical activity in their daily environment.

3. An activity-friendly environment triggers people to engage in a high level of physical activity

In terms of perceived environmental factors, the perception of having easy access to services and stores and seeing many physically active people in their neighborhood were significantly related to the high level of physical activity group. According to the findings of this study, people living with easy access to services and stores were more likely to be both moderately and highly active than those with a low level of physical activity. Perceiving neighbors as being active was significantly associated with a high level of physical activity. This result was consistent with the results from previous studies [44, 45]. According to these findings, people living in a supportive physical environment are important, but it may be insufficient to promote a physically active lifestyle. When the neighborhood is friendly and encourages good social interactions, this can also promote residents to increase physical activity. These results are consistent with paper [46]. In Taiwan, the barrier is a lack of partner, which is an important factor when engaging in physical activity [47]. In general, people like to participate in physical activity with their families, friends, and sports teams. Therefore, government institutions in Taiwan should build supportive environments, such as walking trails, recreational facilities, and bike lanes. In addition, government institutions should encourage the establishment of sports groups and organizations to reinforce the physical activity of people.

## Conclusion

These results can serve as a reference for urban planners and policymakers interested in promoting physical activity for health and can shed light on the associations between the environment and various physical activity levels. Thus, this research could contribute to the development of interdepartmental policies and strategies for HEPA. The rate of HEPA in Taiwanese people was just 30% in 2015; therefore, the Administration of Sport and the Ministry of Education in Taiwan developed a prospective infrastructure construction plan in 2017 to create a friendly environment focused on leisure-time physical activity, to establish a stadium, playground as well as sports facilities in most cities [48]. These strategies will increase people’s physical activity. Therefore, policies and programs should focus on improving continually friendliness and diversity in neighborhoods to facilitate individuals’ transitions from inactive to active lifestyles.

### Limitations

First, the cross-sectional design of this study did not allow for determination of causality. Second, physical activity was measured using the IPAQ showcard version. Therefore, the questions were presented as text and four pictures representing various types of vigorous and moderate physical activity and walking and sitting as well as which can differentiate between domains (e.g., transportation, housework, recreation). However, this aim of study is examined the relationship with level of physical activity and neighborhood environments. Don’t mention the domains of physical activity, because it is already widely known that environmental correlates of physical activity are domain specifics. The participants were asked to accurately recall their levels of engagement in the various intensities of physical activity. Third, consistent with previous studies on physical activity levels, when the participants recalled their levels of engagement in physical activity, they tended to overestimate the time spent on such engagement, and this was particularly evident in the high-activity group [31–33]. However, the IPAQ Showcard Version combined the text questionnaire and figures may increase the reliability and validity. Such systematic biases are likely to have affected the measured associations between neighborhood environments and physical activity. Fourth, self-reported measures of perceived neighborhood environments may not accurately reflect objective measurements; however, these measures are still relevant because actual and perceived neighborhood environments may be independently associated with physical activity [49, 50]. Fifth, only questions regarding neighborhoods surrounding people’s homes and workplaces were asked; the participants were not asked how much time they spent or how long they had lived in their neighborhoods.

## Additional files

Additional file 1: The International Physical Activity Questionnaire Showcard Version. (DOCX 62 kb)

Additional file 2: Perceived Neighborhood Environment Questionnaire. (DOCX 295 kb)

## Abbreviations

HEPA: Health-enhancing physical activity
IPAQ: International physical activity questionnaire
VMWPA: Vigorous, moderate, and walking physical activity
