# Supplementary material for: The perceived neighborhood environment is associated with health-enhancing physical activity among adults: a cross-sectional survey of 13 townships in Taiwan
Source: BMC Public Health. 2019 May 7;19:524. doi: 10.1186/s12889-019-6848-4 (PMC6505307; doi:10.1186/s12889-019-6848-4)
Supplement: Supplementary file 1 — The International Physical Activity Questionnaire Showcard Version. (DOCX 62 kb) [file 12889_2019_6848_MOESM1_ESM.docx]

**Perceived neighborhood environment questionnaire**

Think about the environment in and around your neighborhood by this we mean the area all around your home (or workplace) that you could walk to in 10-15 minutes.

1. Many shops, stores, markets or other places to buy things I need are within easy walking distance of my home. Would you say that you…

| □ Strongly disagree | □ Somewhat disagree | □ Somewhat agree | □ Strongly agree |
| --- | --- | --- | --- |

1. It is within a 10-15 minutes walk to a transit stop (such as bus, train, MRT, or shuttle bus) from my home (or workplace). Would you say that you…

| □ Strongly disagree | □ Somewhat disagree | □ Somewhat agree | □ Strongly agree |
| --- | --- | --- | --- |

1. My neighborhood has several free or low cost recreation facilities, such as schools, parks, green space, open space, square of temple, walking trails, bike paths, recreation centers, stadium, playgrounds, swimming pools, etc. Would you say that you…

| □ Strongly disagree | □ Somewhat disagree | □ Somewhat agree | □ Strongly agree |
| --- | --- | --- | --- |

1. There are sidewalks or storefronts on most of the streets in my neighborhood. Would you say that you…

| □ Strongly disagree | □ Somewhat disagree | □ Somewhat agree | □ Strongly agree |
| --- | --- | --- | --- |

1. The sidewalks in my neighborhood are well maintained (paved, with few cracks) and not obstructed. Would you say that you…

| □ Strongly disagree | □ Somewhat disagree | □ Somewhat agree | □ Strongly agree |
| --- | --- | --- | --- |

1. There are facilities to bicycle in or near my neighborhood, such as special lanes, separate paths or trails, shared use paths for cycles and pedestrians. Would you say that you…

| □ Strongly disagree | □ Somewhat disagree | □ Somewhat agree | □ Strongly agree |
| --- | --- | --- | --- |

1. The crime rate in my neighborhood makes it unsafe to go on walks at night. Would you say that you…

| □ Strongly disagree | □ Somewhat disagree | □ Somewhat agree | □ Strongly agree |
| --- | --- | --- | --- |

1. There are many four-way intersections in my neighborhood. Would you say that you…

| □ Strongly disagree | □ Somewhat disagree | □ Somewhat agree | □ Strongly agree |
| --- | --- | --- | --- |

1. I feel safe when I use the crosswalk to the other side of street. Would you say that you…

| □ Strongly disagree | □ Somewhat disagree | □ Somewhat agree | □ Strongly agree |
| --- | --- | --- | --- |

1. The air quality in my neighborhood is not good. Would you say that you…

| □ Strongly disagree | □ Somewhat disagree | □ Somewhat agree | □ Strongly agree |
| --- | --- | --- | --- |

1. I see many people being physically active in my neighborhood doing things like walking, jogging, cycling, folk dance, tai chi, or playing sports and active games. Would you say that you…

| □ Strongly disagree | □ Somewhat disagree | □ Somewhat agree | □ Strongly agree |
| --- | --- | --- | --- |

1. There are many interesting things to look at while walking in my neighborhood. Would you say you…

| □ Strongly disagree | □ Somewhat disagree | □ Somewhat agree | □ Strongly agree |
| --- | --- | --- | --- |
