# Supplementary material for: The perceived neighborhood environment is associated with health-enhancing physical activity among adults: a cross-sectional survey of 13 townships in Taiwan
Source: BMC Public Health. 2019 May 7;19:524. doi: 10.1186/s12889-019-6848-4 (PMC6505307; doi:10.1186/s12889-019-6848-4)

**The International Physical Activity Questionnaire Showcard Version**

The questions will ask you about the time you spent being physically active in the **last 7 days**. Please answer each question even if you do not consider yourself to be an active person. Please think about the activities you do at work, as part of your house and yard work, to get from place to place, and in your spare time for recreation, exercise or sport.

| **1.** Think about all the **vigorous** activities that you did in the **last 7 days**. **Vigorous** physical activities refer to activities that take hard physical effort and make you breathe much harder than normal. Think only about those physical activities that you did for at least 10 minutes at a time.  **See *Red picture.***  Have you done any of following activities in the last 7 days?  Please tick it. | **2.** Think about all the **moderate** activities that you did in the **last 7 days**. **Moderate** activities refer to activities that take moderate physical effort and make you breathe somewhat harder than normal. Think only about those physical activities that you did for at least 10 minutes at a time.  **See *Yellow picture.***  Have you done any of following activities in the last 7 days?  Please tick it. | | 3. Think about the time you spent **walking** in the **last 7 days**. This includes at work and at home, walking to travel from place to place, and any other walking that you have done solely for recreation, sport, exercise, or leisure.  **See *Green picture.***  Have you done any of following activities in the last 7 days?  Please tick it. |
| --- | --- | --- | --- |
| 1. During the **last 7 days**, on how many days did you do **vigorous** physical activities?   _____**hours per day**  □NO  ***Skip to question 2*** | 1. During the **last 7 days**, on how many days did you do **Moderate** physical activities?   _____**hours per day**  □NO ***Skip to question 3*** | | 1. During the last 7 days, on how many days did you walk for at least 10 minutes at a time?   _____**hours per day**  □NO  ***Skip to question 4*** |
| 1b. How much time did you usually spend doing **vigorous** physical activities on one of those days?  _____**minutes per day** | 2b. How much time did you usually spend doing **Moderate** physical activities on one of those days?  _____**minutes per day** | | 3b. How much time did you usually spend **walking** on one of those days?  _____**minutes per day** |
| **4. Sitting (See Blue picture)** | | **5. sleep** | |
| 1. During the **last 7 days**, how much time did you spend **sitting** on a **week day**? | | 1. During the **last 7 days**, how much time did you spend **sleeping** on a **week day**? | |
| _____**hours and** _____**minutes per day** | | _____**hours and** _____**minutes per day** | |
| 4b. During the **last 7 days**, how much time did you spend **sitting** on a **weekend day**? | | 5b. During the **last 7 days**, how much time did you spend **sitting** on a **weekend day**? | |
| _____**hours and** _____**minutes per day** | | _____**hours and** _____**minutes per day** | |
| During the **last 7 days**, how many days did you spend working?  □**0** □**0.5** □**1** □**1.5** □**2** □**2.5** □**3** □**3.5**□**4** □**4.5** □**5** □**5.5** □**6** □**6.5** □**7天** | | | |


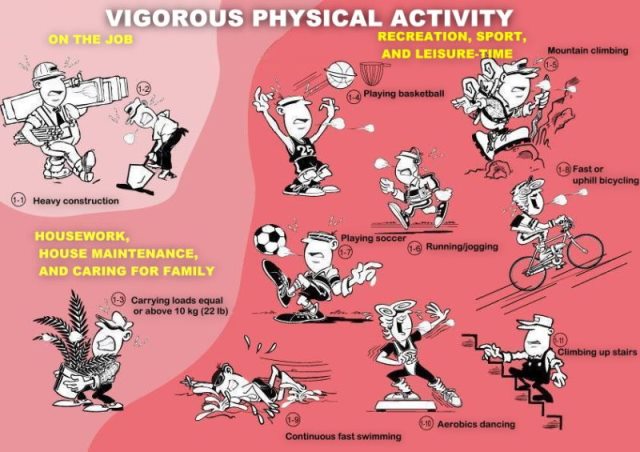

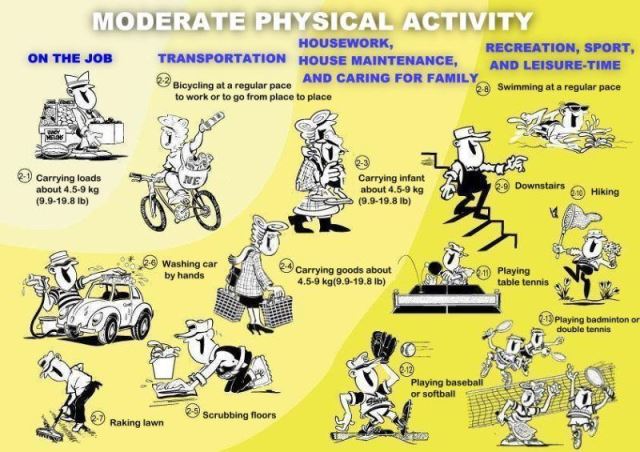

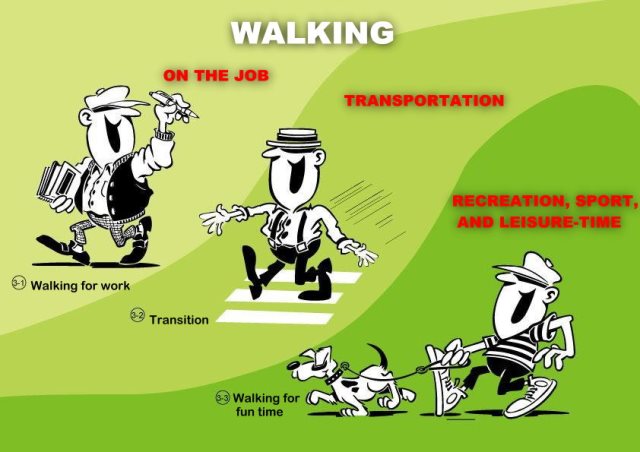

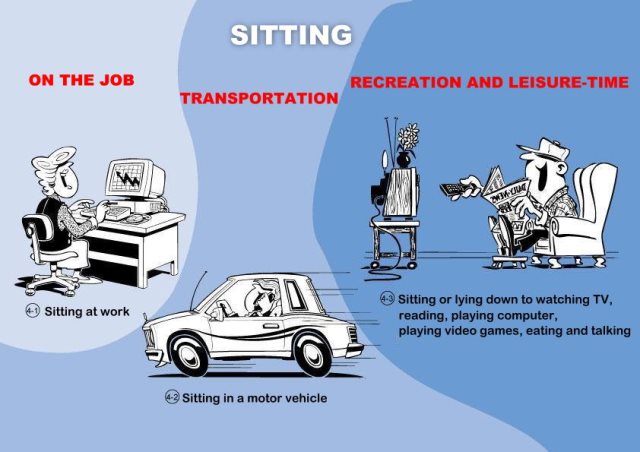

Supplement: Supplementary file 2 — Perceived Neighborhood Environment Questionnaire. (DOCX 295 kb) [file 12889_2019_6848_MOESM2_ESM.docx]
